# Supplementary material for: Is ‘distinctiveness centrality’ actually distinctive? A comment on Fronzetti Colladon and Naldi (2020)
Source: PLoS One. 2024 Apr 16;19(4):e0298666. doi: 10.1371/journal.pone.0298666 (PMC11020676; doi:10.1371/journal.pone.0298666)
Supplement: Attachment — Submitted filename: Letter2.pdf [file pone.0298666.s001.pdf]

Summary of revisions to "Is 'distinctiveness centrality' actually distinctive? A comment on Fronzetti Colladon and Naldi (2020)" (PONE-D-23-34468)

Dear Dr. Kim,

Thank you for obtaining these helpful comments, and for the opportunity to submit a revised manuscript. I describe below how I have addressed each of the reviewers' comments, which I believe have substantially strengthened the manuscript.

1 Reviewer #1

1.1 The paper lacks some relevant references as centrality measures have a vast literature. For example: the survey paper on this topic is also not cited – Centrality measures in complex networks: A survey, by A. Saxena and S. Iyengar

Because this paper is a brief comment on Fronzetti Colladon and Naldi [1], and not a general study of centrality in networks, I have tried to keep the background section very narrowly focused on that topic. However, in the revised version I now end the first paragraph by noting that Fronzetti Colladon and Naldi's work: "*...contributes to the already-vast literature on centrality indices and their relationships to each other [2, 3].*"

The survey paper by Saxena and Iyengar [4] looks very interesting, however it does not appear to be published. I am not sure if *PLOS One* permits citing unpublished pre-prints, however I will defer to the editor for guidance on this. Alternatively, if [4] has been published, I would appreciate if you could point me to the reference.

1.2 At line 65, the author can mention how Fronzetti Colladon and Naldi chose the value of alpha from a wide range for the sake of completeness as now the reader has to go to the original paper to understand it better.

I do not know why Fronzetti Colladon and Naldi chose the values of alpha they consider because they do not explain this in the original paper. I have clarified this in the revision: "*Fronzetti Colladon and Naldi imply that the allowable range of the tuning parameter is $1 \leq \alpha < \infty$, but do not offer an explanation. In their analyses, they examine the performance of D1 for $1 \leq \alpha \leq 15$, but also do not explain why they chose these values [1].*"

1.3 At line 123, in Equ. 4: author proposes to compute alpha based on p. However, it will make alpha vary from $-\infty$ to $+\infty$ and that does not make sense as mentioned by the author earlier. So please add an explanation for that. Please add how such a situation will be handled or controlled.

I have clarified this in the revision: "*In principle, p can take any real value, however in the analyses below I consider only $-1 \leq p \leq 1$ because it is within this range that all three measures perform as identical centrality and power indices.*"

1.4 One main concern is that the results are shown on very small networks or BA networks that are not a good representation of real-world networks having communities. I will suggest adding results for large-scale networks. The authors should also perform experiments on synthetic networks having modular/community structure to verify the applicability.

This paper is a brief comment on Fronzetti Colladon and Naldi [1]. Therefore, I have focused the main text specifically on the networks that [1] use in their analysis. The analyses reported in the main text exactly replicates the analyses reported in the original article, using all the same networks, thereby making the findings directly comparable. [1] did not test their measure in, or make any claims about its performance in, large-scale networks or networks with modular/community structure. Therefore, I do not comment on this issue in the main text.

However, I agree that considering these measures' performance in large-scale networks or networks with modular/community structure is an interesting issue. Therefore, I now investigate this in the supplementary information. Specifically, the supplementary information now compares the three centrality measures in (A) a large 1000-node scale-free network, (B) a large 1000-node small-world network, and (C) a large 1000-node modular network with 10 communities. The paper's conclusions remain unchanged in light of these supplementary analyses.

1.5 The results show the correlation of centrality measure value. However the centrality ranking is different from the centrality measures. In centrality ranking the nodes are ranked based on their centrality value. The author also should study the correlation of centrality ranking.

The reported analyses do study the correlation of centrality ranking, as requested. In the paper, I explain "*Following [1], I then compute the Spearman correlation between D1 and BC, and between D1 and GC, for each p to measure the similarity in node rankings that these measures yield.*" Unlike a Pearson correlation coefficient, the Spearman correlation coefficient measures the correlation between the rank order of two variables (see `https://en.wikipedia.org/wiki/Spearman%27s_rank_correlation_coefficient`.

2 Reviewer #2

2.1 By theoretical and numerical analysis, this paper elaborates that the proposed 'distinctiveness centrality' for node importance is essentially the same as previous one. I think that the reason provided in the paper is sufficient, and the paper is also well organized. Therefore, I would like to recommend that this paper can be accepted in current version.

I am very pleased to hear that you found the argument in the paper sufficient and well organized, and that you have recommended it be accepted.

References

[1] Fronzetti Colladon A, Naldi M. Distinctiveness centrality in social networks. Plos one. 2020;15(5):e0233276. doi:10.1371/journal.pone.0233276.

[2] Valente TW, Coronges K, Lakon C, Costenbader E. How correlated are network centrality measures? Connections. 2008;28(1):16–26.

[3] Borgatti SP, Everett MG. A graph-theoretic perspective on centrality. Social networks. 2006;28(4):466–484. doi:10.1016/j.socnet.2005.11.005.

[4] Saxena A, Iyengar S. Centrality measures in complex networks: A survey. arXiv preprint arXiv:201107190. 2020;.
